# Light-Dependent Nitrate Removal Capacity of Green Microalgae

**DOI:** 10.3390/ijms24010077

**Published:** 2022-12-21

**Authors:** Vaishali Rani, Gergely Maróti

**Affiliations:** 1 Institute of Plant Biology, Biological Research Centre, 6726 Szeged, Hungary; 2 Faculty of Science and Informatics, University of Szeged, 6720 Szeged, Hungary; 3 Department of Water Sciences, University of Public Service, 6500 Baja, Hungary

**Keywords:** *Chlamydomonas*, synthetic wastewater, light color, light intensity, nitrate, nitrate reductase

## Abstract

In the present study, *Chlamydomonas* sp. MACC-216 was used to investigate total nitrate removal in TAP medium with sodium nitrate as the sole nitrogen source under several light conditions made up of permuted combinations of three light colors (referred to as blue, red, and white light) and three light intensities (50 µmol m^−2^ s^−1^, 100 µmol m^−2^ s^−1^, and 250 µmol m^−2^ s^−1^). It was observed that nitrate removal efficiency is influenced by light color as well as light intensity. Additionally, *Chlamydomonas* sp. MACC-216 was cultivated in synthetic wastewater under four light conditions, namely, Blue 250, Blue 125 + Red 125, Red 250, and White 250, where it showed the highest nitrate removal efficiency and nitrate reductase activity under the Blue 125 + Red 125 light condition. To observe the impact of light color on the nitrate removal capacity of *Chlamydomonas* sp. MACC-216, the expression of five genes participating in nitrate transport and reduction (*NRT1*, *NRT2.1*, *NRT2.2*, *NIA*, and *MCP*) was also analyzed; these genes showed the highest expression under the Blue 125 + Red 125 light condition. Based on the above-mentioned findings, the blue + red light combination emerged as a promising light combination for nitrate removal. Hence, our study suggests the importance of the blue + red light combination together with high light intensity, as the optimal light condition for nitrate removal from synthetic wastewater in comparison to other monochromatic lights with high light intensity.

## 1. Introduction

With the increasing global population, there is a surge in industrialization which is responsible for releasing various gaseous, solid, and liquid wastes into the environment. Rising levels of nitrogen and phosphorus in water bodies have been associated with major environmental problems in multiple countries. Nitrate, nitrite, and ammonia represent the main forms of inorganic nitrogen. Nitrite and ammonia are unstable, while nitrate is highly stable making nitrate one of the most common chemical contaminants in water bodies [1]. Nitrate contamination through industrial and domestic wastewater causes harmful algal blooms and rapid eutrophication of natural water ecosystems. Biological methods for wastewater treatment are much more environment-friendly in comparison to physico-chemical decontamination methods [2,3]. Among biological methods, the application of microalgae has emerged as a promising approach for wastewater treatment over the years [4,5,6,7]. Microalgae are capable of utilizing inorganic nutrients such as nitrogen and phosphorus from wastewater as they need them for their photosynthetic/photoheterotrophic/fermentative growth.

The ability of microalgae to grow and propagate in wastewater can be influenced by multiple factors including nutrient concentration, light conditions, pH, temperature, salinity, etc. Light has an essential role in the life cycle of cyanobacteria, algae, and higher plants; the color or wavelength of the light strongly influences their growth [8,9]. Light is essential to microalgae as it helps in the synthesis of vital molecules for growth by the production of ATP and NADPH. Microalgae utilize light for photosynthesis, with photosynthetic light wavelengths ranging from 400 nm to 700 nm. Photosynthetic pigments chlorophyll-a and chlorophyll-b have two major absorption bands: blue or blue-green (450–475 nm) and red (630–675 nm) [10]. Carotenoids represent a group of biological chromophores that play an important role in photosynthesis and have an absorption range between 400 and 550 nm [10]. As absorption bands of photosynthetic pigments lie in the blue and red range of visible light, numerous studies have been carried out to characterize the effects of these two colors on microalgal growth. Several studies have reported the positive effect of red or blue light or both on the biomass yields of *Nannochloropsis* sp., *Scenedesmus* sp., and *Chlamydomonas reinhardtii* [11,12,13]. Biomass productivity of green microalgae has been shown to increase when they are exposed to blue light [11,14,15], whereas other studies have shown a higher growth rate under red light in comparison to blue light [16,17]. In one of the studies, a blue light: red light ratio of 7:3 showed a higher nitrogen removal rate in comparison to solely white, red or blue light in *Scenedesmus* sp. [12]. Most of the studies have focused solely on red or blue light but not their combination, which is why there is still data scarcity on the effect of this particular light combination on various parameters in algae.

Similar to light color, light intensity is another factor that has been shown to play a role in the life cycle of microalgae. Microalgae grown under various light intensities showed differences in growth rate and nutrient removal rate [18,19,20,21]. High light intensity can cause photoinhibition, whereas low light intensity might lead to slow growth; therefore, studies have investigated the optimal light intensity at which maximum growth of the specific microalgae can be achieved. Maltsev et al. indicated that light intensity values are optimal between 26–400 µmol m^−2^ s^−1^ as the maximum growth rate has been observed for different species of microalgae in this light intensity range [9]. Certain algal species such as *Chlamydomonas reinhardtii* cc124 and *Chlorella ohadii* can resist photoinhibition and were shown to grow at light intensities of 3000 µmol m^−2^ s^−1^ and 3500 µmol m^−2^ s^−1^, respectively [22,23]. In the case of nutrient removal, *Chlorella vulgaris* showed an increasing trend in total nitrogen and total phosphorus removal efficiency when the light intensity was increased from 400 µmol m^−2^ s^−1^ to 2000 µmol m^−2^ s^−1^ [24].

The aim behind the present study was to observe whether various light colors and intensities would affect the nitrate removal efficiency of *Chlamydomonas* sp. MACC-216. The specific goal was to determine optimal light color and intensity for efficient nitrate removal by *Chlamydomonas* sp. MACC-216 as this microalga proved in our previous study to be a promising candidate for nitrate removal [25]. The present study investigated the influence of combinations of various light colors and intensities on the nitrate removal efficiency of *Chlamydomonas* sp. MACC-216 grown in tris-acetate-phosphate medium with sodium nitrate as the sole nitrogen source (TAP-N) and synthetic wastewater (SWW). Various light conditions made up of combinations of three light colors (blue, red, and white) and three light intensities (50 µmol m^−2^ s^−1^, 100 µmol m^−2^ s^−1^, and 250 µmol m^−2^ s^−1^) were used to cultivate *Chlamydomonas* sp. MACC-216. *Chlamydomonas* sp. MACC-216 was first grown under each light condition to check for growth and nitrate removal efficiency in TAP-N and then the most efficient light conditions showing maximum growth and nitrate removal capacity were selected for further experiments in SWW. In SWW, *Chlamydomonas* sp. MACC-216 was grown under four different light conditions, namely, Blue 250, Blue 125 + Red 125, Red 250, and White 250, where the numbers 250 and 125 define light intensity in µmol m^−2^ s^−1^. Under the aforementioned four light conditions, various parameters such as growth, nitrate removal efficiency, nitrate reductase activity and expression of genes involved in nitrate transport and reduction were investigated in *Chlamydomonas* sp. MACC-216.

## 2. Results

### 2.1. Effect of Various Light Conditions on the Growth of Chlamydomonas sp. MACC-216

**Figure 1:**
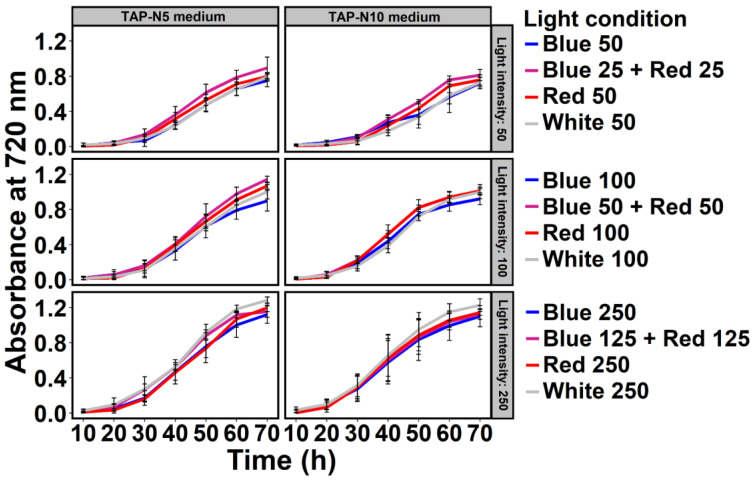
Growth of *Chlamydomonas* sp. MACC-216 under various light conditions in TAP-N5 and TAP-N10 media. Numbers 25, 50, 100, 125, and 250 mentioned in the figure represent light intensity. The unit of light intensity is µmol m^−2^ s^−1^.

The growth of *Chlamydomonas* sp. MACC-216 was mainly influenced by the light intensity in TAP-N5 (TAP with 5 mM nitrate) and TAP-N10 media (TAP with 10 mM nitrate) (Figure 1). At 50 µmol m^−2^ s^−1^ light intensity, *Chlamydomonas* sp. MACC-216 grew slower in comparison to 100 µmol m^−2^ s^−1^ or 250 µmol m^−2^ s^−1^ light intensity. It was also observed that up to 250 µmol m^−2^ s^−1^, *Chlamydomonas* sp. MACC-216 growth is directly proportional to light intensity as an increase in the microalgal growth was observed when light intensity was increased from 50 µmol m^−2^ s^−1^ to 250 µmol m^−2^ s^−1^. However, no major difference was observed between the growth curves of *Chlamydomonas* sp. MACC-216 grown in TAP-N5 and in TAP-N10 media at any light condition.

### 2.2. Effect of Various Light Conditions on the Nitrate Removal Capacity of Chlamydomonas sp. MACC-216

**Figure 2:**
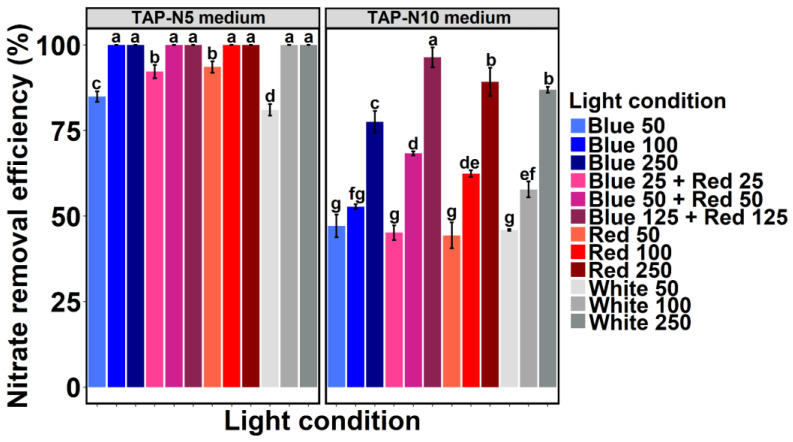
Nitrate removal efficiency of *Chlamydomonas* sp. MACC-216 under various light conditions in TAP-N5 and TAP-N10 media. Numbers 25, 50, 100, 125, and 250 mentioned in the figure represent light intensity. The unit of light intensity is µmol m^−2^ s^−1^. Error bars represent standard deviations. Tukey’s-test was done for each nitrate concentration separately. Lowercase letters signify statistical differences (*p* < 0.05) as determined by Tukey’s-test.

*Chlamydomonas* sp. MACC-216 grown under TAP-N5 medium showed the highest nitrate removal efficiency under Blue 25 + Red 25 and Red 50 light conditions at 50 µmol m^−2^ s^−1^ light intensity (Figure 2). Least nitrate removal efficiency was observed in the case of the White 50 light condition. Furthermore, nitrate removal efficiency was increased when the light intensity was increased from 50 µmol m^−2^ s^−1^ to 100 µmol m^−2^ s^−1^. However, when light intensity was increased to either 100 µmol m^−2^ s^−1^ or 250 µmol m^−2^ s^−1^, no significant difference in nitrate removal efficiency was observed among various light conditions. When *Chlamydomonas* sp. MACC-216 was cultivated in TAP-N10 medium at 50 µmol m^−2^ s^−1^ light intensity, no significant difference in nitrate removal efficiency was observed among the various light conditions (Blue 50, Blue 25 + Red 25, Red 50, and White 50). An increase in the light intensity from 50 µmol m^−2^ s^−1^ to 250 µmol m^−2^ s^−1^ led to significantly higher nitrate removal efficiency. Thus, *Chlamydomonas* sp. MACC-216 grown under Blue 250 removed more nitrate than *Chlamydomonas* sp. MACC-216 grown under Blue 50 and so on. A clear role of light intensity was observed in total nitrate removal as higher nitrate removal efficiency was observed at higher light intensity. Moreover, *Chlamydomonas* sp. MACC-216 cultivated in TAP-N10 medium under Blue 125 + Red 125 showed the highest significant nitrate removal efficiency in comparison to any other light condition. *Chlamydomonas* sp. MACC-216 removed 9.64 mM nitrate when grown under Blue 125 + Red 125 light condition (Table S1). At 250 µmol m^−2^ s^−1^ light intensity, the least nitrate removal efficiency was observed under Blue 250 light condition.

### 2.3. Growth, Nitrate Removal Efficiency and Nitrate Reductase Activity in SWW

**Figure 3:**
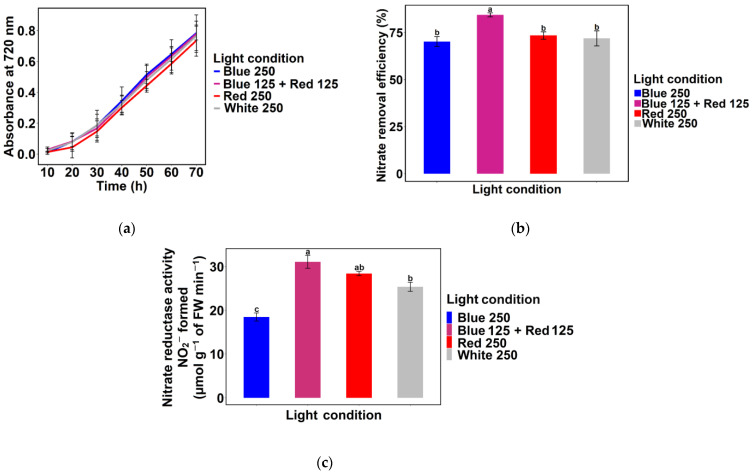
*Chlamydomonas* sp. MACC-216 under various light conditions in SWW: (**a**) Growth; (**b**) Nitrate removal efficiency; (**c**) Nitrate reductase activity. FW: Fresh weight. Error bars represent standard deviations. Lowercase letters signify statistical differences (*p* < 0.05) as determined by Tukey’s-test.

For experiments to be carried out in SWW, 250 µmol m^−2^ s^−1^ light intensity was chosen because *Chlamydomonas* sp. MACC-216 showed the highest growth and nitrate removal efficiency in TAP-N5 and TAP-N10 media at this light intensity. No significant difference was observed among the growth curves of *Chlamydomonas* sp. MACC-216 grown under Blue 250, Blue 125 + Red 125, Red 250, and White 250 light conditions (Figure 3a). However, *Chlamydomonas* sp. MACC-216 showed slower growth in SWW (Figure 3a) in comparison to TAP-N5 and TAP-N10 media (Figure 1) at 250 µmol m^−2^ s^−1^ light intensity. Even in SWW, *Chlamydomonas* sp. MACC-216 showed highest nitrate removal efficiency under Blue 125 + Red 125 light condition (Figure 3b). *Chlamydomonas* sp. MACC-216 grown under Blue 125 + Red 125 light condition removed 4.22 mM nitrate, whereas *Chlamydomonas* sp. MACC-216 grown under Red 250 and White 250 light conditions removed 3.67 mM and 3.60 mM, respectively (Table S2). *Chlamydomonas* sp. MACC-216 grown under the Blue 250 light condition removed the least nitrate (3.51 mM).

Nitrate reductase activity of *Chlamydomonas* sp. MACC-216 was quantified to investigate the influence of various light conditions on this enzyme activity. We observed that *Chlamydomonas* sp. MACC-216 grown under Blue 125 + Red 125 light had significantly the highest nitrate reductase activity in comparison to any other light conditions used in the study (Figure 3c). Nitrate reductase of *Chlamydomonas* sp. MACC-216 grown under Blue + Red 125 light condition produced 31.09 µmol g^−1^ of FW (fresh weight) min^−1^ NO_2_^−^, whereas *Chlamydomonas* sp. MACC-216 grown under Blue 250, Red 250, and White 250 light conditions produced 18.47, 28.41 and 25.4 µmol g^−1^ of FW min^−1^ NO_2_^−^, respectively (Table S2).

### 2.4. Expression of Genes Involved in Nitrate Transport and Reduction

**Figure 4:**
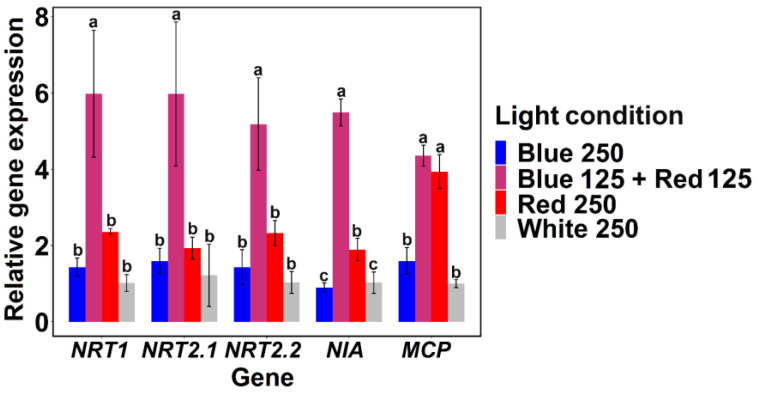
Relative gene expression of *NRT1*, *NRT2.1*, *NRT2.2*, *NIA*, and *MCP* genes in *Chlamydomonas* sp. MACC-216 grown under various light conditions in SWW. Error bars represent standard deviations. Tukey’s-test was done for each gene separately. Lowercase letters signify statistical differences (*p* < 0.05) as determined by Tukey’s-test.

The expression of genes participating in nitrate transport and reduction in *Chlamydomonas* sp. MACC-216 was investigated. Five genes were selected namely, *NRT1*, *NRT2*.1, *NRT2*.2, *NIA*, and *MCP* where *NRT1*, *NRT2*.1, *NRT2*.2 code for nitrate and/or nitrite transporters, *NIA* codes for nitrate reductase, and *MCP* codes for Moco (Molybdenum cofactor) carrier protein. Expression of *NRT1*, *NRT2*.1, *NRT2.2*, *NIA*, and *MCP* was significantly higher in *Chlamydomonas* MACC-216 grown under the Blue 125 + Red 125 light condition (Figure 4 and Table S3). After the Blue 125 + Red 125 light condition, *NRT1*, *NRT2*.1, *NRT2.2*, *NIA*, and *MCP* genes showed high expression under the Red 250 light condition.

### 2.5. Growth and Nitrate Removal Efficiency of Chlorella sp. MACC-38 and Chlorella sp. MACC-360 in SWW

**Figure 5:**
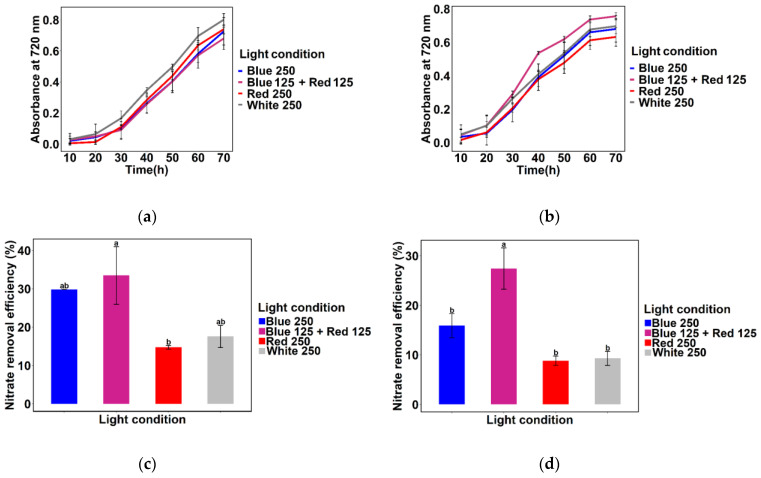
Growth of *Chlorella* sp. MACC-38 (**a**) and *Chlorella* sp. MACC-360 (**b**) under various light conditions in SWW. Nitrate removal efficiency of *Chlorella* sp. MACC-38 (**c**) and *Chlorella* sp. MACC-360 (**d**) under various light conditions in SWW. Error bars represent standard deviations. Lowercase letters in (**c**,**d**) signify statistical differences (*p* < 0.05) as determined by Tukey’s-test.

*Chlorella* sp. MACC-38 and *Chlorella* sp. MACC-360 were grown in SWW under Blue 250, Blue 125 + Red 125, Red 250, and White 250 light conditions to check whether these two microalgae would also perform like *Chlamydomonas* sp. MACC-216 in SWW. No major difference was observed among the growth curves of *Chlorella* sp. MACC-38 grown under various light conditions (Figure 5a), whereas *Chlorella* sp. MACC-360 grew better under the Blue 125 + Red 125 light condition in comparison to other light conditions (Figure 5b). Significant high nitrate removal efficiency was observed under Blue 125 + Red 125 light condition in *Chlorella* sp. MACC-360 only (Figure 5c,d). Even under Blue 125 + Red 125 light condition, both microalgae removed less nitrate in comparison to *Chlamydomonas* sp. MACC-216. *Chlorella* sp. MACC-38 and *Chlorella* sp. MACC-360 removed 1.67 mM and 1.37 mM nitrate, respectively, whereas *Chlamydomonas* sp. MACC-216 removed 4.22 mM nitrate under the same light conditions (Tables S2 and S4). Both microalgae removed the least nitrate under Red 250 and White 250 light conditions.

## 3. Discussion

The present investigation was undertaken to elucidate the effects of combinations of various light colors (blue, red, and white) and intensities (50 µmol m^−2^ s^−1^, 100 µmol m^−2^ s^−1^, and 250 µmol m^−2^ s^−1^) on the nitrate removal capacity of *Chlamydomonas* sp. MACC-216. Since the light spectrum has been known to affect microalgae, we decided to check its effect on the total nitrate removal by microalgae. Additionally, we also wanted to study whether the combination of blue and red light will enhance the growth and nitrate removal efficiency of *Chlamydomonas* sp. MACC-216 as absorption bands of photosynthetic pigments lie in the blue and red range of visible light. So far, numerous studies have shown the effects of different light colors or wavelengths and intensities on the growth of various microalgae [11,12,19,21,26]; however, only a few studies have utilized a blue + red light color combination [12,27]. When cultivated in TAP-N5 and TAP-N10 media, the growth of *Chlamydomonas* sp. MACC-216 only seemed to be influenced by light intensity as it increased with increasing light intensity, where the maximum light intensity used was 250 µmol m^−2^ s^−1^ (Figure 1). Similarly, a continuous increase in the growth of *Chlorella* sp. 800 has been reported with increasing light intensity until 500 µmol m^−2^ s^−1^ [18]. Light intensity has been known to play a major role in the lifecycle of microalgae by affecting the photosynthesis of microalgae and consequently their growth. In general, the light intensity can increase the growth rate of microalgae if it is increased up to a certain point, which depends on specific microalgae species. Too high a light intensity can lead to photoinhibition whereas too low a light intensity can lead to a decrease in the growth of the microalgae. In the case of nitrate removal efficiency, we observed that this variable was affected by light color, light intensity, and concentration of nitrate. At 5 mM nitrate concentration, we did not see any major significance in nitrate removal under various light conditions (Figure 2). However, at 10 mM nitrate concentration, *Chlamydomonas* sp. MACC-216 removed the highest amount of nitrate under the Blue 125 + Red 125 light condition (Table S1). Our findings of high nitrate removal under blue + red light are consistent with the study of Kim et al., where *Scenedesmus* sp. has been shown to remove nitrate at a higher rate when grown under blue + red light combination in comparison to solely blue, red, and white light [12]. Moreover, *Chlamydomonas* sp. MACC-216 showed increased nitrate removal efficiency in TAP-N5 and TAP-N10 media with increasing light intensity (Figure 2). *Chlamydomonas reinhardtii* strain 21 gr demonstrated high nitrate removal when the light intensity was increased from 400 µmol m^−2^ s^−1^ to 1000 µmol m^−2^ s^−1^, which is consistent with our observations [28]. High nitrate removal at high light intensity may be due to the increased demand for nitrogen in algae to repair light-induced damage in photosystems caused by high light intensity. Furthermore, we decided to use continuous light for our study as previous studies have confirmed better growth and nitrate removal under continuous light [29,30]. In one study, *Chlorella kessleri* under continuous illumination and 12 h:12 h (Light:Dark) lighting showed nitrate removal efficiency of 19% and 9%, respectively [29]. The same study also showed higher cell concentrations under continuous illumination in comparison to 12 h:12 h (Light:Dark) lighting. Similarly, in another study, an increase in algal biomass of a mixed algae culture (*Chlamydomonas reinhardtii, Scenedesmus rubescens, and Chlorella vulgaris*) was observed under continuous illumination in contrast to 12 h:12 h (Light:Dark) lighting [30]. The decrease in cell biomass observed under the 12 h:12 h (Light:Dark) condition could be caused by biomass loss through respiration [29].

*Chlamydomonas* sp. MACC-216 grown in SWW under Blue 250, Blue 125 + Red 125, Red 250, and White 250 light conditions showed slower growth and lower nitrate removal efficiency in comparison to *Chlamydomonas* sp. MACC-216 grown in TAP-N5 and TAP-N10 media. *Chlamydomonas* sp. MACC-216 cultivated in SWW under Blue 125 + Red 125 showed the highest nitrate removal efficiency in comparison to any other light condition (Figure 3b). A similar observation was noticed for *Chlorella* sp. MACC-38 and *Chlorella* sp. MACC-360 (Figure 5c,d). The high nitrate reductase activity of the *Chlamydomonas* sp. MACC-216 grown under the Blue 125 + Red 125 light condition correlated with its high nitrate removal efficiency in SWW (Figure 3b,c). Likewise, low nitrate reductase activity of the *Chlamydomonas* sp. MACC-216 grown under the Blue 250 light condition correlated with its low nitrate removal efficiency under the Blue 250 light condition. In some higher plants such as etiolated pea and tissues of maize, light-stimulated de novo synthesis of nitrate reductase was shown to be caused by red light via the phytochrome system [31]. The pigment phytochrome is known to be photoconverted from inactive Pr (red light absorbing phytochrome) to physiologically active Pfr (far-red-light absorbing phytochrome) by red light [31,32]. Figueroa suggested the presence of two types of receptors in red algae *Corallina elongate*, namely phytochrome and b-light receptor for red and blue light colors, respectively [33]. Aparicio and Quiñones showed the importance of blue + red light through their study on *Monoraphidium braunii* [34]. They observed that when *M. braunii* cells were grown under only red light, the nitrate uptake was slower in comparison to when grown under red + blue light continuously and red + intermittent blue light. Moreover, it has been shown that blue light is necessary for in vivo activation of inactive nitrate reductase in *Chlamydomonas reinhardtii* [35]. To conclude, perhaps red light is required for the biosynthesis of nitrate reductase and blue light is probably needed for nitrate uptake and activation of the nitrate reductase enzyme. This might be the reason behind the high nitrate removal efficiency and nitrate reductase activity under the Blue 125 + Red 125 light condition in our study.

To investigate whether different light conditions are modulating the transcription of genes involved in nitrate uptake and reduction, the expression of five genes, namely *NRT1*, *NRT2.1*, *NRT2.2*, *NIA*, and *MCP* was investigated by quantitative reverse transcription polymerase chain reaction (RT-qPCR). *NRT1*, *NRT2.1*, and *NRT2.2* code for nitrate and/or nitrite transporters, *NIA* codes for nitrate reductase, and *MCP* codes for Moco (Molybdenum cofactor) carrier protein. Three families of transporters namely, NRT1, NRT2, and NAR1 have been shown to play a role in the nitrate and/or nitrite transport in *Chlamydomonas* [36,37]. Transporters NRT1, NRT2, and NAR1 consist of one, six and six transporters, respectively [36]. The NRT1 transporter encoded by a single *NRT1* gene in *Chlamydomonas* belongs to the nitrate peptide transporter family (NPF) [37,38]. NRT1 transporter is well studied in *Arabidopsis* as a dual affinity nitrate transporter, but there is a lack of information about this transporter in *Chlamydomonas* [34,35]. NRT2 transporters belong to the nitrate/nitrite porter (NNP) family which further belongs to the major facilitator superfamily (MFS) [37]. Six genes (*NRT2.1*-*6*) code for the transporters in the NRT2 transporters family in *Chlamydomonas* [36]. Both NRT2.1 and NRT2.2 transporters need NAR2 protein to be fully functional; thus, NRT2.1 together with NAR2 makes transporter system I bi-specific for nitrate and nitrite, while NRT2.2 together with NAR2 makes nitrate specific transporter system II [36,37]. Other transporters (NRT2.3-6) in the NRT2 family are low-affinity nitrate transporters [36,39]. NAR1 transporters only play a role in nitrite and bicarbonate transportation; hence, we decided to choose *NRT1*, *NRT2.1* and *NRT2.2* genes from nitrate/nitrite transporter families for expression study as these three seem to play the role of the main nitrate transporters. We observed the highest expression of all these three genes under the Blue 125 + Red 125 light condition in comparison to other light conditions (Figure 4). This high expression correlates with high nitrate reductase activity and high nitrate removal as observed from our above-mentioned results. Therefore, it is clear from our study that light color affects the transport of nitrate and thereby increases or decreases the expression of genes encoding nitrate transporters in the algae.

Nitrate reductase coded by the *NIA* gene plays a role in the reduction of nitrate to nitrite in the cytoplasm. *Chlamydomonas* algae have a single gene encoding nitrate reductase in their genome [36,37]. In eukaryotes, nitrate reductase is known to be a homodimeric protein consisting of 100–120 kDa subunits and each subunit contains five structurally distinct domains: molybdenum-molybdoprotein (Mo-MPT), dimer interface, cytochrome-b, FAD binding domain and NADPH binding domain [40]. Moco carrier protein (MCP) encoded by the *MCP* gene is known to play a role in the biosynthesis and transfer of the molybdenum cofactor to the nitrate reductase which is necessary for the enzyme to function [36,41]. Similar to the nitrate transporters, the expression of *NIA* and *MCP* was also found to be light color dependent as both genes were highly expressed under the Blue 125 + Red 125 light condition in comparison to other light conditions (Figure 4).

Although the growth curves of *Chlamydomonas* MACC-216 grown under various light conditions showed no major difference, *Chlamydomonas* MACC-216 grown under the Blue 125 + Red 125 light condition showed the highest nitrate removal efficiency and the highest expression of genes involved in nitrate transport and reduction. This non-correlation between the growth and expression of different genes under various light conditions could be due to the regulation of gene expression at the translational or post-translational level in *Chlamydomonas* MACC-216.

The present study confirmed the greater role of the Blue 125 + Red 125 light condition in nitrate removal at the gene expression level. Although in the present study, *Chlamydomonas* sp. MACC-216 was cultivated in SWW under the above-mentioned light condition, the next step could be to use this light condition to perform total nitrogen and phosphorus removal studies in real wastewater collected from various industrial plants. Furthermore, transcriptome studies could be performed for *Chlamydomonas* sp. MACC-216 grown under the Blue 125 + Red 125 light condition. This analysis would be useful to observe the detailed transcriptional reorganization taking place inside *Chlamydomonas* sp. MACC-216 under the Blue 125 + Red 125 light condition which can pave the way for a better understanding of this condition affecting microalgae.

## 4. Materials and Methods

### 4.1. Microalga Strain and Growth Media

*Chlamydomonas* sp. MACC-216 strain used for the current research study was provided by the Mosonmagyaróvár Algae Culture Collection (MACC) and was initially maintained in TAP medium consisting of 2.42 g L^−1^ Tris base, 0.374 g L^−1^ NH_4_Cl, 0.204 g L^−1^ MgSO_4_·7H_2_O, 0.066 g L^−1^ CaCl_2_·2H_2_O, 0.287 g L^−1^ K_2_HPO_4_, 0.142 g L^−1^ KH_2_PO_4_, 0.049 g L^−1^ Na_2_EDTA·2H_2_O, 0.039 g L^−1^ ZnSO_4_·7H_2_O, 0.011 g L^−1^ H_3_BO_3_, 0.007 g L^−1^ MnCl_2_·4H_2_O, 0.008 g L^−1^ FeSO_4_·7H_2_O, 0.002 g L^−1^ CoCl_2_·6H_2_O, 0.002 g L^−1^ CuSO_4_·5H_2_O, 0.001 g L^−1^ (NH_4_)_6_Mo_7_O_24_·4H_2_O, and 1 mL L^−1^ CH_3_COOH with pH maintained at 7. The final concentration of CH_3_COOH in TAP medium was 16.8 mM. The microalga was maintained at 25 °C under white light with a light intensity of 50 µmol m^−2^ s^−1^ with continuous shaking at 180 rpm in a regime of 16:8 light-dark periods.

For further experiments, a modified TAP medium (TAP-N) was prepared by substituting different concentrations of sodium nitrate as the nitrogen source instead of ammonium chloride. TAP-N5 and TAP-N10 media were prepared by adding 5 mM (424.97 mg L^−1^), and 10 mM (849.94 mg L^−1^), respectively, of sodium nitrate. In addition, 0.001 g L^−1^ (NH_4_)_6_Mo_7_O_24_·4H_2_O was replaced with 0.006 g L^−1^ of Na_2_MoO_4_·2H_2_O in TAP-N medium so that there is no other nitrogen source other than sodium nitrate.

Synthetic wastewater (SWW) used in the present study was prepared according to the procedure mentioned in “OECD guidelines for testing chemicals” after slight modifications [42]. To 1 L of distilled water, 1.6 g peptone, 1.1 g meat extract, 0.425 g NaNO_3_ (5 mM), 0.07 g NaCl, 0.04 g CaCl_2_·2H_2_O, 0.02 g MgSO_4_·7H_2_O, and 0.28 g K_2_HPO_4_ were added, and the pH was set at 7.5.

### 4.2. Growth Conditions and Measurement

*Chlamydomonas* sp. MACC-216 was grown in TAP-N5 and TAP-N10 media under various combinations of light colors (or wavelengths) and light intensities. In total, 12 light conditions made up of combinations of three light colors (blue, red, and white) and three light intensities (50 µmol m^−2^ s^−1^, 100 µmol m^−2^ s^−1^, and 250 µmol m^−2^ s^−1^) were selected to cultivate *Chlamydomonas* sp. MACC-216 (Table 1). *Chlamydomonas* sp. MACC-216 was grown under each light condition in Multi-Cultivator MC-1000-OD (Photon Systems Instruments) with a continuous light source for 70 h. The absorbance was automatically measured at 720 nm at a gap of 10 h each.

For SWW, only four light conditions were selected namely, Blue 250, Blue 125 + Red 125, Red 250, and White 250 (Table 1) and the absorbance was measured at 720 nm similarly as mentioned above. Furthermore, *Chlorella* sp. MACC-38 and *Chlorella* sp. MACC-360 were also grown under the above-mentioned conditions to examine their growth and nitrate removal capacity in SWW.

### 4.3. Nitrate Measurement

Nitrate estimation was performed at 0 h and 70 h using the salicylic acid method [43]. Briefly, to 10 µL of each sample, 40 µL of 5% (*w*/*v*) salicylic acid in concentrated H_2_SO_4_ was added. Samples were kept at room temperature for 30 min and then 950 µL of 8% (*w*/*v*) NaOH in water was added followed by cooling down of samples to room temperature. The absorbance was determined at 410 nm using a Hidex microplate reader.

The nitrate removal efficiency was calculated using the following equation:

(1) $$R=\left(1-\frac{Cf}{Ci}\right)\times 100$$

where *R* is the nitrate removal efficiency (%), *Ci* and *Cf* are the initial and final concentrations of nitrate (mM) in the growth medium.

### 4.4. Nitrate Reductase Activity

For nitrate reductase activity, *Chlamydomonas* sp. MACC-216 was grown for 48 h under Blue 250, Blue 125 + Red 125, Red, and White 250 light conditions in SWW. After 48 h, 10 mL of culture was collected from each light condition and centrifuged at 4000 rpm for 5 min at 4 °C. Afterwards, supernatants were discarded, and pellets were dissolved in ice-cold 0.1 M phosphate buffer (pH 7.0) followed by sonication at 0.8 cycle, 90% amplitude for 30 s on ice. After sonication, centrifugation was performed at 17,000 rpm for 10 min at 4 °C and supernatants were collected. In a fresh microcentrifuge, to 400 µL of supernatant, 500 µL of 10 mM KNO_3_, 40 µL of 5mM β-Nicotinamide adenine dinucleotide, reduced disodium salt hydrate (Sigma-Aldrich, St. Louis, MO, USA) and 60 µL of Milli-Q water were added followed by incubation at 25 °C for 30 min. Afterwards, 500 µL of 10 mM zinc acetate was added to stop the reaction followed by centrifugation at 10,000 rpm for 5 min. The supernatant was collected for nitrite estimation by Griess reagent assay as described by Giovannoni et al. [44]. For the Griess reagent, two solutions were prepared namely, 1% (*w*/*v*) sulphanilamide (Sigma-Aldrich, St. Louis, MO, USA) in 5% (*v*/*v*) phosphoric acid and 0.1% (*w*/*v*) N-(1-naphthyl)ethylenediamine dihydrochloride (NED) (Sigma-Aldrich, St. Louis, MO, USA) in water. Both solutions are stable for several months at 4 °C in darkness. To 100 µL of supernatant, first, 100 µL sulphanilamide solution was added followed by incubation at room temperature for 10 min. Then, 100 µL of NED solution was added followed by further incubation at room temperature for 10 min. After incubation, absorbance was measured at 540 nm in a Hidex microplate reader.

### 4.5. Quantification of Genes Expression

For RNA isolation, *Chlamydomonas* sp. MACC-216 was grown for 70 h under Blue 250, Blue 125 + Red 125, Red 250, and White 250 light conditions in SWW. 1 mL of microalgae sample was collected from each light condition for RNA isolation. RNA isolation was performed using TRIzol^TM^ reagent (Thermo Fisher Scientific, Waltham, MA, USA) by following manual instructions. cDNA was prepared from isolated RNA using RevertAid First Strand cDNA Synthesis Kit (Thermo Fisher Scientific, Waltham, MA, USA) by following manual instructions.

To perform quantitative reverse transcription polymerase chain reaction (RT-qPCR), first primers were designed for two reference genes (GAPDH and beta 1 tubulin) and five genes involved in nitrate transportation and reduction (Table 2). Accession numbers for these genes were obtained from Fernandez and Galvan and Sanz-Luque et al. [36,37] and sequences were obtained from the NCBI database (https://www.ncbi.nlm.nih.gov/). RT-qPCR was performed with 50 ng of cDNA, 500 nM each of forward and reverse primer, 5 µL of SYBR green PCR master mix (Applied Biosystems, Thermo Fisher Scientific, Waltham, MA, USA) and nuclease-free water to a final concentration of 10 µL.

Analysis of the results obtained by RT-qPCR was undertaken using Pfaffl method [45]. For this, primer efficiency was calculated for all primer pairs. White 250 light condition was selected as a control for the analysis. The expression of each gene is provided in terms of the relative gene expression which is based on the expression ratio of a target gene versus the reference genes.

### 4.6. Statistical Analysis

Rstudio version 1.2.5019 was used to perform the statistical analyses. All statistical analyses in the current study were performed using one-way analysis of variance (ANOVA) followed by Tukey’s test which was used to detect significant differences among various light conditions, where *p* < 0.05 was considered a significant difference. All experiments were carried out in replicates of 4.

## 5. Conclusions

*Chlamydomonas* sp. MACC-216 was used to study the effects of various combinations of three light colors (blue, red, and white) and three light intensities (50 µmol m^−2^ s^−1^, 100 µmol m^−2^ s^−1^, and 250 µmol m^−2^ s^−1^) on its nitrate removal efficiency in TAP-N medium. A role of light intensity as well as light color was observed on the nitrate removal efficiency of the studied microalgae. Furthermore, *Chlamydomonas* sp. MACC-216 was cultivated under four light conditions namely, Blue 250, Blue 125 + Red 125, Red 250, and White 250 in SWW, where it showed the highest nitrate removal efficiency and nitrate reductase activity under the Blue 125 + Red 125 light condition in comparison to any other light conditions. Relative gene expression values of five genes involved in nitrate transport and reduction were also observed to be the highest under the Blue 125 + Red 125 light condition. Therefore, it is to be concluded that blue + red light combination together with high light intensity represents optimal conditions for the applied green microalgae to efficiently remove nitrate from wastewater.

## Figures and Tables

**Table 1:** Light conditions for the cultivation of *Chlamydomonas* sp. MACC-216 in TAP-N and SWW.

Light Condition	Growth Medium	Description
Blue 50	TAP-N5 and TAP-N10	Blue light with 50 µmol m^−2^ s^−1^ light intensity
Blue 25 + Red 25	TAP-N5 and TAP-N10	Blue light with 25 µmol m^−2^ s^−1^ light intensity + Red light with 25 µmol m^−2^ s^−1^ light intensity
Red 50	TAP-N5 and TAP-N10	Red light with 50 µmol m^−2^ s^−1^ light intensity
White 50	TAP-N5 and TAP-N10	White light with 50 µmol m^−2^ s^−1^ light intensity
Blue 100	TAP-N5 and TAP-N10	Blue light with 100 µmol m^−2^ s^−1^ light intensity
Blue 50 + Red 50	TAP-N5 and TAP-N10	Blue light with 50 µmol m^−2^ s^−1^ light intensity + Red light with 50 µmol m^−2^ s^−1^ light intensity
Red 100	TAP-N5 and TAP-N10	Red light with 100 µmol m^−2^ s^−1^ light intensity
White 100	TAP-N5 and TAP-N10	White light with 100 µmol m^−2^ s^−1^ light intensity
Blue 250	TAP-N5, TAP-N10, and SWW	Blue light with 250 µmol m^−2^ s^−1^ light intensity
Blue 125 + Red 125	TAP-N5, TAP-N10, and SWW	Blue light with 125 µmol m^−2^ s^−1^ light intensity + Red light with 125 µmol m^−2^ s^−1^ light intensity
Red 250	TAP-N5, TAP-N10, and SWW	Red light with 250 µmol m^−2^ s^−1^ light intensity
White 250	TAP-N5, TAP-N10, and SWW	White light with 250 µmol m^−2^ s^−1^ light intensity

**Table 2:** List of genes and their primers used for real-time PCR.

Gene Name	NCBI Accession Number	Primer (5′→3′)
Nitrate Transporter (*NRT1*)	XM_043061965	F: AGGCTCTGCCCCTGATAGA R: CCTCCCATCACATTGCAGA
Nitrate Transporter (*NRT2.1*)	Z25438	F: TGAGAAGCCAGCCACAGTAA R: AAGCAAATCCAGGACAGGTG
Nitrate Transporter (*NRT2.2*)	Z25439	F: CCATCTTCGGCCTTATGAAC R: CGTTAGCGAGTTGCTGACCT
Nitrate reductase (*NIA*)	AF203033	F: AGCCGTTGACTTTGACCATG R: GCATGTTCTCCTCCTTGCG
Molybdenum cofactor (moco) carrier protein (*MCP*)	AY039706	F: CATGGCTGGATCTTGCTGAC R: CAGGAAGGACACCGATCGT
Glyceraldehyde-3-phosphate dehydrogenase (GAPDH)	L27669	F: ATTGGCCGCCTGGTTATG R: GGTCTTGTGGACCGAGTCAT
Beta 1 tubulin	M10064	F: CGCATGATGCTGACCTTCT R: GTCCAGGACCATGCACTCAT

F and R denote forward and reverse primer, respectively.

## Data Availability

The data presented in this study are available on request from the corresponding author.

## Supplementary Materials

The following supporting information can be downloaded at: https://www.mdpi.com/article/10.3390/ijms24010077/s1.
