# Supplementary material for: Light-Dependent Nitrate Removal Capacity of Green Microalgae
Source: Int J Mol Sci. 2022 Dec 21;24(1):77. doi: 10.3390/ijms24010077 (PMC9820755; doi:10.3390/ijms24010077)
Supplement: Supplementary file 1 [file ijms-24-00077-s001.zip › ijms-2097188-supplementary.pdf]

International Journal of
Molecular Sciences

MDPI

Table S1. Total nitrate removal by *Chlamydomonas* sp. MACC-216 under various light conditions in TAP-N5 and TAP-N10 media. Values are represented as mean ± standard deviation.

Light condition	Total nitrate removal in TAP-N5 medium (mM)	Total nitrate removal in TAP-N10 medium (mM)
Blue 50	4.25 ± 0.07^{c}	4.71 ± 0.33^{g}
Blue 100	5 ± 0.0^{a}	5.27 ± 0.07^{fg}
Blue 250	5 ± 0.0^{a}	7.76 ± 0.31^{c}
Blue 25 + Red 25	4.61 ± 0.09^{b}	4.52 ± 0.21^{g}
Blue 50 + Red 50	5 ± 0.0^{a}	6.83 ± 0.05^{d}
Blue 125 + Red 125	5 ± 0.0^{a}	9.64 ± 0.3^{a}
Red 50	4.68 ± 0.08^{b}	4.44 ± 0.37^{g}
Red 100	5 ± 0.0^{a}	6.24 ± 0.1^{de}
Red 250	5 ± 0.0^{a}	8.92 ± 0.41^{b}
White 50	4.05 ± 0.08^{d}	4.60 ± 0.02^{g}
White 100	5 ± 0.0^{a}	5.78 ± 0.23^{ef}
White 250	5 ± 0.0^{a}	8.69 ± 0.08^{b}

Superscript lowercase letters signify statistical differences ($P < 0.05$) as determined by Tukey's-test. Tukey's-test was done for each nitrate concentration separately.

Table S2. Values of total nitrate removal and nitrate reductase activity under various light conditions in SWW. Values are represented as mean ± standard deviation.

Light condition	Total nitrate removal (mM)	Nitrate reductase activity [NO_2^- formed (μmol g^{-1} of FW min^{-1})]
Blue 250	3.51 ± 0.13[b]	18.47 ±0.91[c]
Blue 125 + Red 125	4.22 ± 0.05[a]	31.09 ± 1.48[a]
Red 250	3.67 ± 0.09[b]	28.41 ± 0.48[ab]
White 250	3.60 ± 0.19[b]	25.4 ± 1.04[b]

FW: Fresh weight
Superscript lowercase letters signify statistical differences (P <0.05) as determined by Tukey's-test.

Table S3. Values of the relative gene expression of *NRT1, NRT2.1, NRT2.2, NIA,* and *MCP* in *Chlamydomonas* sp. MACC-216 grown under various light conditions in SWW. Values are represented as mean ± standard deviation.

Gene	Relative gene expression			
	**Blue 250**	**Blue 125 + Red 125**	**Red 250**	**White 250**
NRT1	1.43 ± 0.23[b]	6.0 ± 1.7[a]	2.36 ± 0.08[b]	1.01 ± 0.22[b]
NRT2.1	1.59 ± 0.32[b]	6.0 ± 1.9[a]	1.93 ± 0.29[b]	1.21 ± 0.80[b]
NRT2.2	1.43 ± 0.46[b]	5.18 ± 1.21[a]	2.32 ± 0.32[b]	1.02 ± 0.29[b]
NIA	0.90 ± 0.11[c]	5.49 ± 0.35[a]	1.89 ± 0.29[b]	1.02 ± 0.28[c]
MCP	1.6 ± 0.34[b]	4.35 ± 0.28[a]	3.93 ± 0.44[a]	1.0 ± 0.10[b]

Superscript lowercase letters signify statistical differences (P <0.05) as determined by Tukey's-test.

Table S4: Total nitrate removal by *Chlorella* sp. MACC-38 and *Chlorella* sp. MACC-360 under various light conditions in SWW. Values are represented as mean ± standard deviation.

Light condition	Total nitrate removal (mM)	
	Chlorella sp. MACC-38	*Chlorella* sp. MACC-360
Blue 250	1.50 ± 0.01[ab]	0.79 ±0.12[b]
Blue 125 + Red 125	1.67 ± 0.38[a]	1.37 ± 0.20[a]
Red 250	0.73 ± 0.02[b]	0.44 ± 0.05[b]
White 250	0.88 ± 0.14[ab]	0.46 ± 0.07[b]

Superscript lowercase letters signify statistical differences ($P < 0.05$) as determined by Tukey's-test.
